# Rice false smut pathogen: implications for mycotoxin contamination, current status, and future perspectives

**DOI:** 10.3389/fmicb.2024.1344831

**Published:** 2024-03-20

**Authors:** Lei Zhou, Mustansar Mubeen, Yasir Iftikhar, Hongxia Zheng, Zhenhao Zhang, Junli Wen, Raja Asad Ali Khan, Ashara Sajid, Manoj Kumar Solanki, Muhammad Aamir Sohail, Ajay Kumar, Ehab El Sayed Massoud, Liezhong Chen

**Affiliations:** ^1^State Key Laboratory for Managing Biotic and Chemical Threats to the Quality and Safety of Agro-products, Institute of Agro-product Safety and Nutrition, Zhejiang Academy of Agricultural Sciences, Hangzhou, China; ^2^Department of Plant Pathology, College of Agriculture, University of Sargodha, Sargodha, Pakistan; ^3^College of Plant Protection, Hainan University, Haikou, China; ^4^Department of Life Sciences and Biological Sciences, IES University, Bhopal, Madhya Pradesh, India; ^5^Plant Cytogenetics and Molecular Biology Group, Institute of Biology, Biotechnology and Environmental Protection, Faculty of Natural Sciences, University of Silesia in Katowice, Katowice, Poland; ^6^National Key Laboratory of Plant Molecular Genetics, Center for Excellence in Molecular Plant Sciences, Institute of Plant Physiology and Ecology, Chinese Academy of Sciences, Shanghai, China; ^7^Amity University of Biotechnology, Amity University, Noida, India; ^8^Biology Department, Faculty of Science and Arts in Dahran Aljnoub, King Khalid University, Abha, Saudi Arabia

**Keywords:** rice, smut, RFS, mycotoxin, infection process

## Abstract

Rice serves as a staple food across various continents worldwide. The rice plant faces significant threats from a range of fungal, bacterial, and viral pathogens. Among these, rice false smut disease (RFS) caused by *Villosiclava virens* is one of the devastating diseases in rice fields. This disease is widespread in major rice-growing regions such as China, Pakistan, Bangladesh, India, and others, leading to significant losses in rice plantations. Various toxins are produced during the infection of this disease in rice plants, impacting the fertilization process as well. This review paper lightens the disease cycle, plant immunity, and infection process during RFS. Mycotoxin production in RFS affects rice plants in multiple ways, although the exact phenomena are still unknown.

## Introduction

**Figure 1:**
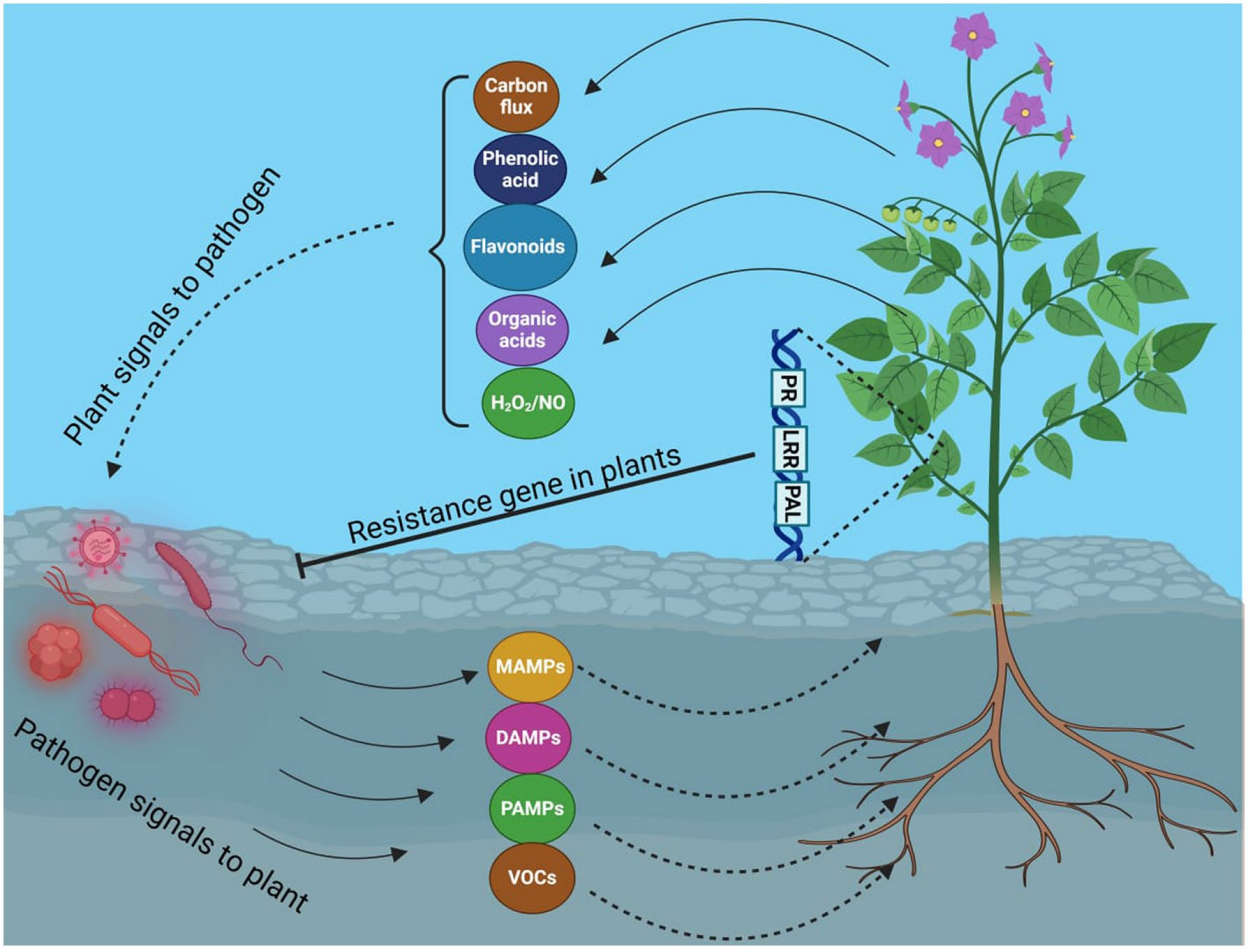
Molecular interaction between host plant and pathogen.

Crop production is vital for food security, and rice production, like other staple food crops, is crucial for ensuring it. The security of the rice supply is not only an economic concern but also a key indicator of political and social stability. Recently, several minor diseases in rice have escalated into serious issues. Rice false smut (RFS) disease, threatening yield and grain quality, is one such disease. RFS has been reported to occur more frequently throughout most major rice-growing regions, including China, India, and the USA (Sun et al., 2020). The widespread use of hybrid rice types, mostly RFS-vulnerable, is thought to have contributed to the rise of this disease. The ascomycete fungal pathogen *Villosiclava virens* (anamorph: *Ustilaginoidea virens* [Cooke] Takahashi), which mainly infects rice blossoms and turns them into RFS balls, is the cause of rice false smut disease (RFS). RFS balls start small before steadily expanding and encasing the floral components. Like many other diseases, false smut appeared as an outbreak in different epidemic years globally (Ladhalakshmi et al., 2012; Snehlata and Kumar, 2015). Recently, it has been attributed that false smut contributes to severe yield losses and disease incidence. The estimated yield losses in China were 158.6 million kg each year from 2008 to 2016 (Lu et al., 2015). Disease incidence increases in several cultivars such as Presidios in Louisiana (Harrell, 2020), with several other susceptible cultivars, such as CL172, CLXL745, Gemini 241 CL, and PLV01, being susceptible to false smut pathogens (Harrell, 2020). Remarkable economic losses were reported even at very low levels of disease incidence. The first balls were discovered to be smooth, slightly flattened, and covered by a thin membrane (Wang et al., 2019), exploding with chlamydospores at this point. When there is a significant temperature difference between day and night in autumn, RFS balls produce sclerotia. The only observable symptom of RFS disease is the RFS ball, leading to significant losses in production and quality due to the presence of RFS balls and increased sterility of kernels surrounding them. The two mycotoxins that RFS balls produce, ustiloxin and ustilaginoidin, harm humans and animals and pose serious health risks by contaminating rice and straws (Dangi et al., 2020). This can cause serious health risks, which may be fatal for humans. Ustiloxin, for instance, inhibits the development of the skeleton and the assembly of microtubules in eukaryotic cells, resulting in kidney and liver damage in mice. In some rice panicles in the paddy field, RFS balls appear randomly and are invariably gathered during harvest (Atia, 2004). The rate of disease spread varies within and between areas, thought to be worse near drainage systems. RFS disease outbreaks frequently coincide with rainfall during the rice booting and heading stages with epidemics varying significantly between varieties, fields, and seasons. This disease is becoming very significant in rice-growing areas these days. RFS disease has a novel host-pathogen interaction mechanism (Figure 1), with the pathogen invading the stigma, styles, and young ovary of each spikelet, blocking the formation of mature pollen and turning them into yellow, olive green, or blackish spore balls, causing rice grain discoloration (Bag, 2010; Fan et al., 2016). The mycotoxins produced by the RFS pathogen react with human metabolism, plants, and animals, posing significant dangers to both (Fan et al., 2016; Wang et al., 2017; Sun et al., 2020). This review will accumulate data showing the mechanism of action of these mycotoxins, and knowledge about them will be reviewed and analyzed.

## Origin and geographical distribution

A majority of rice-growing regions now recognize RFS, caused by the Clavicipitaceous fungus *Ustilaginoidea virens*, also known as *Villosiclava virens*, as one of the most severe rice diseases (Wang et al., 2019). Formerly classified as a mild illness due to its erratic spread and initial location in India’s Tamil Nadu State’s Tirunelveli region, this disease has rapidly spread in major rice-growing areas over the past 20 years. This rapid spread is attributed to extensive planting of high-yield rice cultivars and hybrids, improper application of nitrogenous fertilizer, and global warming. It has been discovered in about one-third of rice cultivation areas. Nearly all countries that produce rice have reported cases of false smut caused by Ustilaginoidea virens, including Australia, Italy, Bangladesh, Philippines, Peru, Myanmar, Fiji, China, Colombia, Japan, Thailand, USA, Bolivia, Brazil, Sri Lanka, Ghana, Indonesia, Ivory Coast, Panama, Nigeria, Pakistan, Sudan, Tanzania, Trinidad, Venezuela, Vietnam, and Zambia, as well as in some American states (Laha et al., 2017). Yield losses due to RFS have been recorded to be more than 75%.

## Economic losses due to rice false smut

Globally, false smut outbreaks occur during epidemic years (Kumari and Kumar, 2015). False Smut (FS) disease causes 0.01–8.6% rice yield losses. In recent years, false smut has been linked to higher incidence and yield losses. From 2008 to 2016, yield losses in China were recorded as 158.6 million kilograms annually by Lu et al. (2018). Depending on weather and rice cultivar., false smut of rice can result in yield losses of 3 to 70% (Baite et al., 2021).

## Pathogen biology

False smut-infected plants produce smut balls at the maturity level, which are greenish-black in color. Sclerotia and chlamydospores are overwintering fungal spores produced by false smut pathogens that overwinter in rice seeds and soil (Fan et al., 2016; Sun et al., 2020). Moreover, this fungus infects not only various types of grasses but also many weed plants such as *Panicum tenellum, Panicum trypheron, Digitaria marginate, Echinochloa crusgalli*, and *Imperata cylindrica* (Schneider and Groth, 2018; Sun et al., 2020). They also act as alternative host plants of this pathogen; hence, they can act as reservoirs of the inoculum in the offseason and the crop season. However, the sclerotia are in the form of horseshoes. Seclerotia are the sexual stage of fungi that can remain dormant for many months (Wang et al., 2019). The sclerotia germinate to separate the stroma under proper moisture, light, and temperature conditions, creating asci-containing ascospores.

For ascospores to contribute to the initial infections of rice, they produce secondary conidia on the surfaces of the false smut balls, thick-walled chlamydospores develop throughout the asexual cycle (Fu et al., 2012). Between seasons, the chlamydospores serve as essential inoculum sources. Secondary conidia cause rice false smut disease that the chlamydospores create, ascospores. Alternative hosts like weeds (*Digitaria marginata*, *Panicum trypheron*, *Echinochola crus Galli*, and *Imperata cylindria*) may also play a role in the life cycle of *U. virens* in addition to rice, with uncommon infections being reported. Whether sclerotia or chlamydospores are the most significant primary inoculum in the field is still debatable, despite advances in our understanding of *U. virens* life cycle. Both sclerotia and chlamydospores have been proven capable of lasting more than ten months in laboratory or outdoor circumstances. Sclerotia and chlamydospores could thus both serve as the main inoculum in the field.

Recent research has revealed that sclerotia can be formed to a significant extent in a variety of geographical areas, including temperate and subtropical zones (Yong et al., 2018). After a 2-to-5-month period of dormancy, the sclerotia begin to germinate and produce ascospores in the presence of light. Before and after rice planting, ascospores are typically trapped in rice paddy fields, showing that sclerotia can overwinter successfully and consistently produce ascospores. Chlamydospores were only discovered in the rice paddy fields when disease symptoms started to manifest. The rice false smut fungus belongs to order Hypocreales and family *Clavicipitaceae*. RFS causes poor yield and leads to turning the rice grain black in color and moving to milking. It also causes spikelet sterility (Dhua et al., 2015). Infected grain are double in diameter of the normal size of the grains. Initially, these were yellow and later turned black (Quintana et al., 2016). In the stages of infection, a small number of grains were infected, but in severe infection, 100% of the field was infected (Ladhalakshmi et al., 2012). This pathogen produces huge amounts of mycotoxins, such as Ustilaginoidins and Ustiloxins (Zhou et al., 2012). These toxins suppress the shoot and root growth of the rice plant (Wang et al., 2017).

## Symptoms

Rice false smut disease, often called green smut, is frequently used to describe an abundant crop. The only visible symptom is the replacement of rice grains by false smut balls or ball-shaped fungal mycelia (Fan et al., 2016). Sclerotia typically appear on the false smut balls in autumn. Although the pathogens begin to affect rice during panicle growth, symptoms do not appear until after flowering. The fungus then grows on top of the spikelet. When grains mature, they all turn into yellowish smut balls that later turn yellowish-orange, green, and greenish-black. When smut balls rupture, powdery, dark green spores are released. False smut directly affects grain yield and quality by replacing some or all kernels with spore masses (Khanal et al., 2023). Symptoms are easily visible on the outermost side of the rice kernels. Whereas symptoms are detectable at the late maturity level or during the harvest level. The characteristic symptoms of false smut are velvety, globose spore balls replacing the rice kernels. Powdery chlamydospores mass are produced (Schneider and Groth, 2018; Sun et al., 2020). The color of immature smut balls is orangish-yellow. However, mature smut balls are greenish-black. The inner organs of flowers are replaced by the fungal mycelia of false smut (Tanaka et al., 2008; Tang et al., 2013). Sclerotia is produced at the sexual stage of fungi, while chlamydospores are asexual spores. Sclerotia can germinate at a high level for up to five years and can cause long-lasting infection in field conditions (Yong et al., 2018). After the germination of sexual and asexual spores, conidia are produced, acting as a primary inoculum. The yield losses depend on rice cultivars and weather conditions. Almost 3 to 70% yield losses were reported due to false smut of rice (Baite et al., 2021). The ratio of rice cultivars infected with false smut disease is not restricted. However, false smut fungi produce mycotoxins such as ustiloxins and ustilaginoidins (Wang et al., 2019). These mycotoxins have carcinogenic effects not only on human health but also on animal health. They become affected by the consumption of straws and grains of rice contaminated with the false smut pathogen (Wang et al., 2019; Sun et al., 2020).

## Morphological characterization of *Ustilaginoidea virens*

Sixty-three isolates were grown on PSA for 21 days at 26 ± 1°C to examine their cultural traits (size, color, texture, growth type, and sporulation) (Bag et al., 2021). The final measurements of size, texture, and color were noted for every isolate on the 28th day. The growth pattern of mycelial growth varied, ranging from extremely slow, slow, and moderate to quick, and the color altered based on the maturity period. As a result, the rate of growth was variable. Ten isolates were less than 25 mm, 37 were between 25 and 40 mm, and 16 were more than 40 mm among the 63 total. *U. virens* mycelial development on PSA initially ranged from off-white to white. The isolates’ colonies also varied in texture, ranging from fluffy raised to compact, cottony raised. Most isolates (36) had compact colonies, 19 had fluffy raised colonies, and the remaining eight had cottony grown colonies. Chlamydospores developed at the colony’s edge or center. The spore maturity time varied as well. As a result, after 21 days, the color of the isolate changed from yellow to greenish-black to whitish-yellow. Hegde et al. (2000) and Ladhalakshmi et al. (2012) specified the pathogen’s morphological features after 28 days. Manu et al. (2017) reported the observations on the pathogen’s growth on the solid medium, including colony color, colony diameter, growth type, and mycelia dry weight after 30 days, by measuring the radial mycelial growth at 10-day intervals.

## Genetic diversity of *Ustilaginoidea virens*

One of the rare fungal infections for which there is little to no study data, particularly regarding population structure and genetic diversity, is rice false smut, or *U. virens*. Jia et al. (2015) employed SSR markers in China to perform a genetic analysis of the population structure of *V. virens*, a teleomorph of the false smut pathogen. To define evolutionary relationships, find potential donors for marker-assisted selection, and manage the disease effectively with the suitable fungicides, a thorough understanding of the genetic diversity and population structure analysis of plant pathogens is essential (Ciampi et al., 2011; Marulanda et al., 2014). Furthermore, understanding evolutionary adaptability and its potential to outpace host resistance requires temporal and spatial information on genetic diversity and pathogen population structure (McDonald and Linde, 2002; Ciampi et al., 2011). Tan et al. (2008) reported that most *V. virens* strains collected from different portions of Hunan province were genetically similar. Higher genetic similarity across isolates from distinct fields indicates that selection of *V. virens* isolates was more heavily influenced by geographic variables. RAPD and single nucleotide polymorphisms were used to examine 110 isolates from various Chinese provinces. The isolates clustered based on their place of origin, and there was more genetic diversity between the populations than within the populations (Wang et al., 2014). The geographical area greatly influenced the genetic variability of *U. virens* compared to the rice cultivars (Sun et al., 2013; Yu et al., 2013; Wang et al., 2014; Jia et al., 2015). At least 193 putative effectors are involved in predicting the interaction between the host-pathogen interaction. Several genes are responsible for the pathogenicity of the pathogen. Eighteen putative effectors depress the plant’s HR (hypersensitive response) (Sun et al., 2013; Fan et al., 2016). These eighteen genes were significantly expressed during the rice field infection (Zhang et al., 2014; Fan et al., 2016).

Moreover, this pathogen has more genes involved in producing several toxins and secondary metabolites. A few genes are involved in polysaccharide degradation, nutrient uptake, and metabolism compared to *Fusarium graminearum* and *M. oryzae*. This was observed due to the analysis of several genomes of the *U. virens* (Zhang et al., 2014). Some genes are involved in the peptide synthetase of nonribosomal and synthetase gene clusters. They play a pivotal role in the production of mycotoxins (Zhang et al., 2014; Fu et al., 2015; Lu et al., 2015; Meng et al., 2015). The study of transcriptome responses between susceptible host (HXZ) as well as resistance host (IR28) of rice. The comparative analysis is done against *U. virens*. It was reported that many different genes are involved in hormone and secondary metabolism. It also contains genes responsible for peroxidases and flavin-containing monooxygenases (Yang et al., 2014; Fan et al., 2016). The initial data regarding genetic variation and population composition of *U. virens* isolates from India. The large region of eastern and northeastern India produced a large number of isolates, some of which came from relatively different rice fields, adding to the isolates’ variability. All *U. virens* populations showed a modest level of genetic variation, with a similarity of over 60%, and some influence from geography, according to the study (Bag et al., 2021). Comprehensive knowledge of the diversity and population structure of *U. virens*, rice false smut disease management strategies and the usage of rice lines tolerant to the illness might be developed.

## Infection process

**Figure 2:**
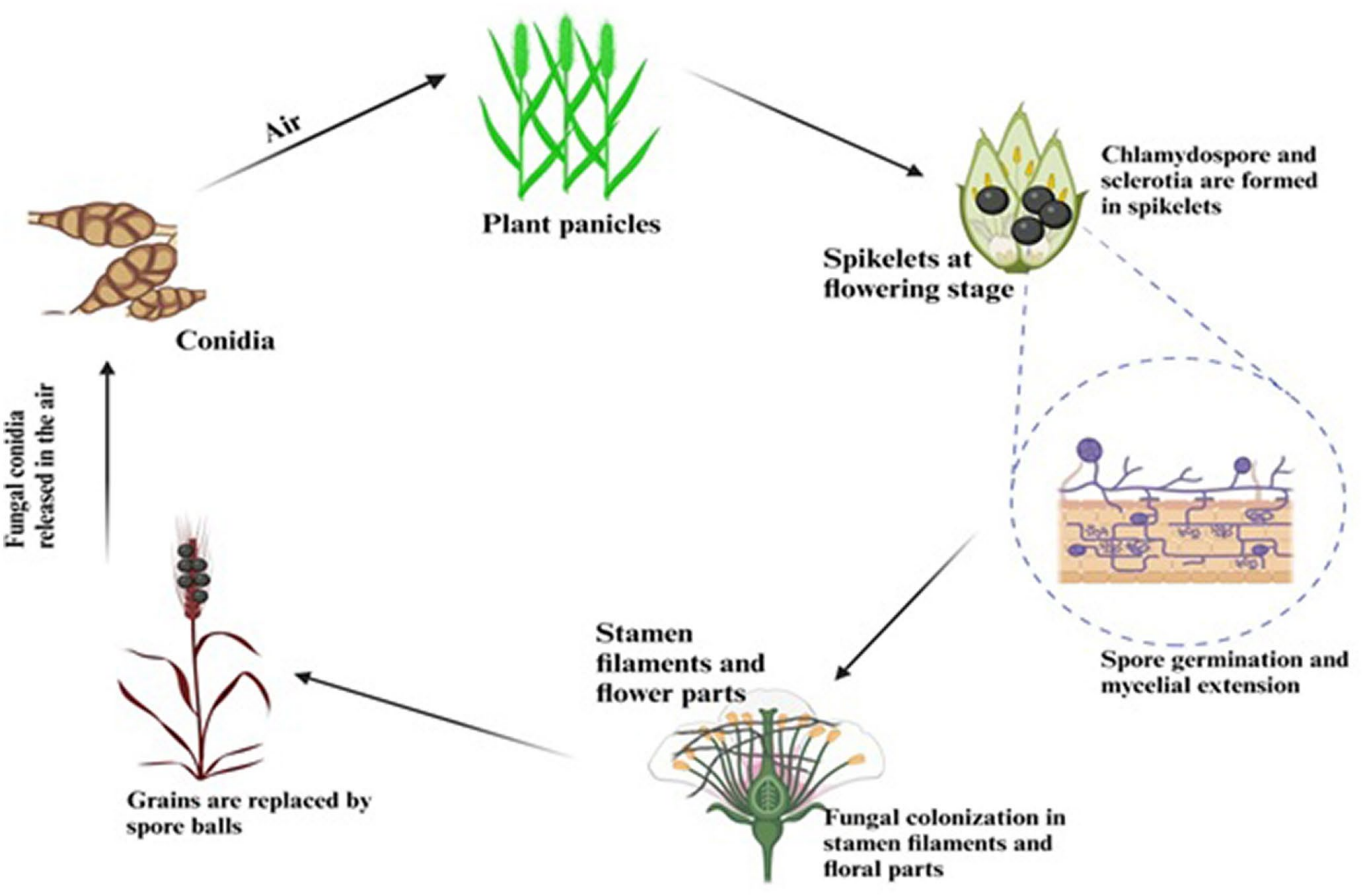
Life cycle showing the infection process of RFS.

**Figure 3:**
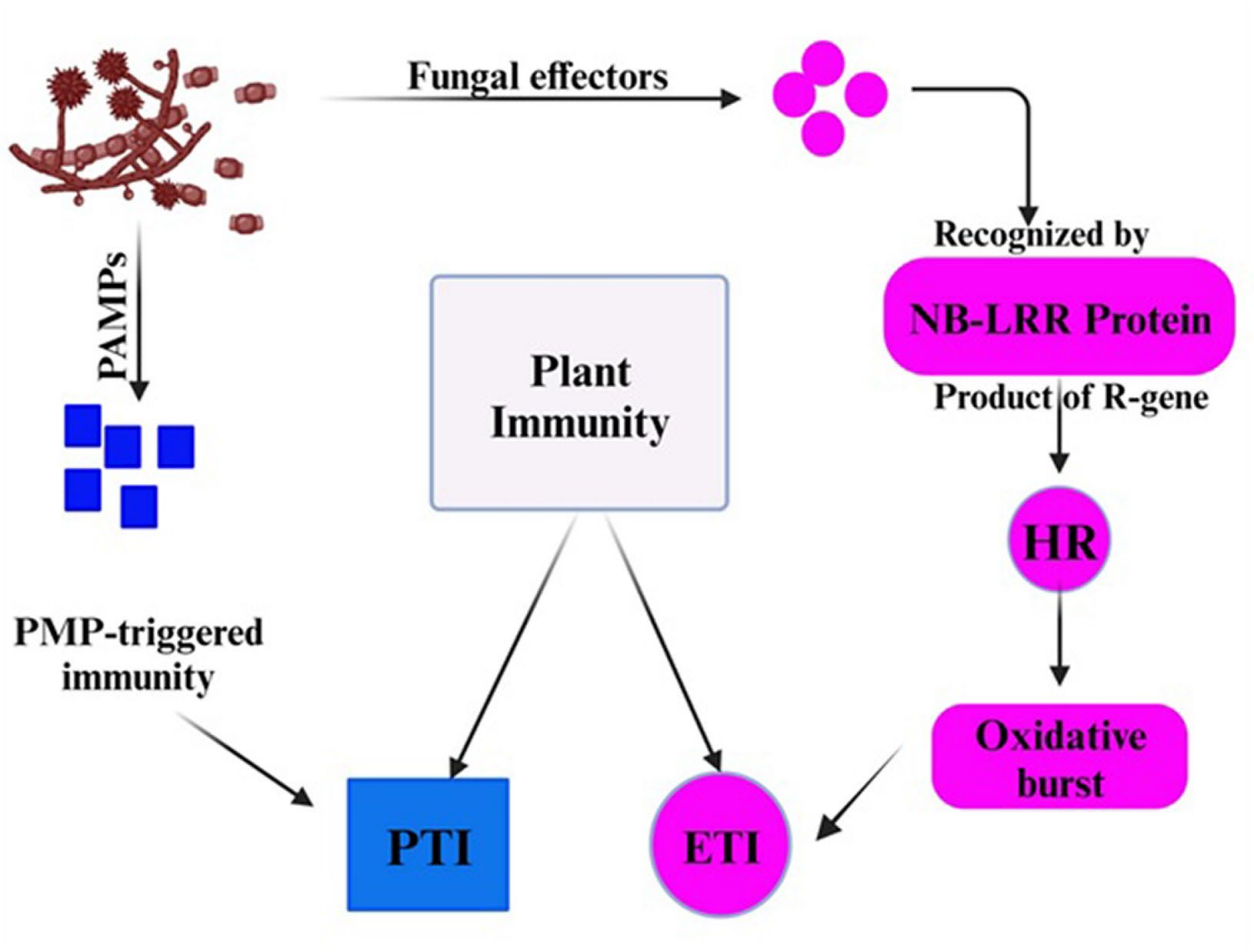
The plant showed immunity in response of fungal effectors.

Although the false smut disease cycle is not yet clear, the chlamydospore germination infection process is still in question. Rice fields have a chance of infection at the booting level. The infection arises from both ascospores and chlamydospore germination, which are produced from the sclerotia. Under favorable conditions, conidia are produced by the fungi upon maturation of the chlamydospores. Spines are prominent on the surface. Additionally, the fungus infects the spikelets at the flowering stage and colonizes the rice, leading to male sterility (Qin et al., 2013). Smut balls are formed on the rice panicle. This pathogen is sporadic (Ashizawa et al., 2012; Nessa et al., 2015). Therefore, the fungi were isolated by Baite and Sharma (2015) from the diseased spikelet on various types of cultural media such as rice yeast dextrose agar (RYDA) and potato dextrose agar (PDA), and potato sucrose agar (PSA). The pathogen growth was observed on the PSA (potato sucrose agar) medium. The colony of the pathogen was creamy white, with a diameter of 40 mm. The growth of the fungi was observed at pH 6 and temperature of 27°C after 30 days of incubation. The life cycle of RFS shows that the conidia are airborne, and sclerotia are found at the spikelet stage of the rice where the spores germinate, leading to the extension of mycelium (Figure 2). The lack of experimental support from cytological analyses has led to a protracted discussion regarding the pathogen infection process. The lack of a reliable and repeatable inoculation process has been one of the key factors. The inoculation procedure has gradually improved in recent years. A study was conducted to use the methodology with a slight modification of a low-temperature treatment (2 days at 20°C) to obtain highly infected spikelets (Hu et al., 2020). This considerably reduced the necessary work and allowed for the acquisition of sufficient numbers of infected spikelets for cytological study. Whole balls might be examined throughout different phases of development in serial sections with numerous false smut balls at various stages (Tang et al., 2013). It has been established that the principal infection sites are the top portions of the three filaments between the lodicules and the ovary. This scarcity of infection-friendly locations could be why inoculation and natural infection are challenging processes. Previous studies demonstrated that inoculation was necessary during the booting stage and that infection establishment after the panicles headed was exceedingly challenging (Jiehui et al., 2022). This might be connected to the filament’s structure and physiological status. Following fertilization, rice pistils will absorb a significant amount of nutrients and photosynthesis products to produce grains. After flowering, the filaments will grow from the spikelets and eventually perish (Kumar et al., 2020). Therefore, when pathogen hyphae were evident on the surfaces of anthers, stigmas, and ovaries, the pistil or its stigmas and ovary have been thought to be the infection sites for the false smut pathogen. The primary infection taking place on the filaments was a surprising discovery. The filaments seem too fragile and transient to support the pathogen’s growth, which needs a lot of nutrients (Khan et al., 2023). The filaments often extend quickly and synchronously with the enlargement of the lodicules to push the anthers out of the lemmas during anthesis in cereal plants. Extension during filament elongation is only possible in the epidermis and one or two layers of sub-epidermal cells. As a result, its cell adhesion and organization must be distinct and weak, which could make it vulnerable to pathogen attack. The lodicule or the bases of stigmata may be invaded by the fungus with a low probability, but the extent of the hyphae was constrained. This suggests that the filament tissue or its surface is considerably more delicate than stigmas and lodicules. The two tiny entities known as lodicules can grow considerably at anthesis, but their vascular tissue does not elongate. It was shown that pathogen hyphae cannot penetrate vascular tissue, which may prevent them from obtaining enough nutrients for ball formation. Even though there were plenty of pathogen hyphae near the ovaries, the pathogen never entered the ovaries. The filaments may, therefore, be the sole primary infection sites, according to this. The lodicule or the bases of stigmata may be invaded by the fungus with a low probability, but the extent of the hyphae was constrained. This suggests that the filament tissue or its surface is considerably more delicate than stigmas and lodicules. The two tiny entities known as lodicules can grow considerably at anthesis, but their vascular tissue did not elongate. It was shown that pathogen hyphae cannot penetrate vascular tissue, which may prevent them from obtaining enough nutrients for ball formation. Even though there were plenty of pathogen hyphae close to the ovaries, the pathogen never entered the ovaries. The filaments may, therefore be the sole primary infection sites, according to this. The infection process also depends on NB-LRR Protein that produces the HR response and PMP immunity is triggered when the fungal effectors are recognized by NB-LRR protein (Figure 3).

## Nutrient acquisition

**Figure 4:**
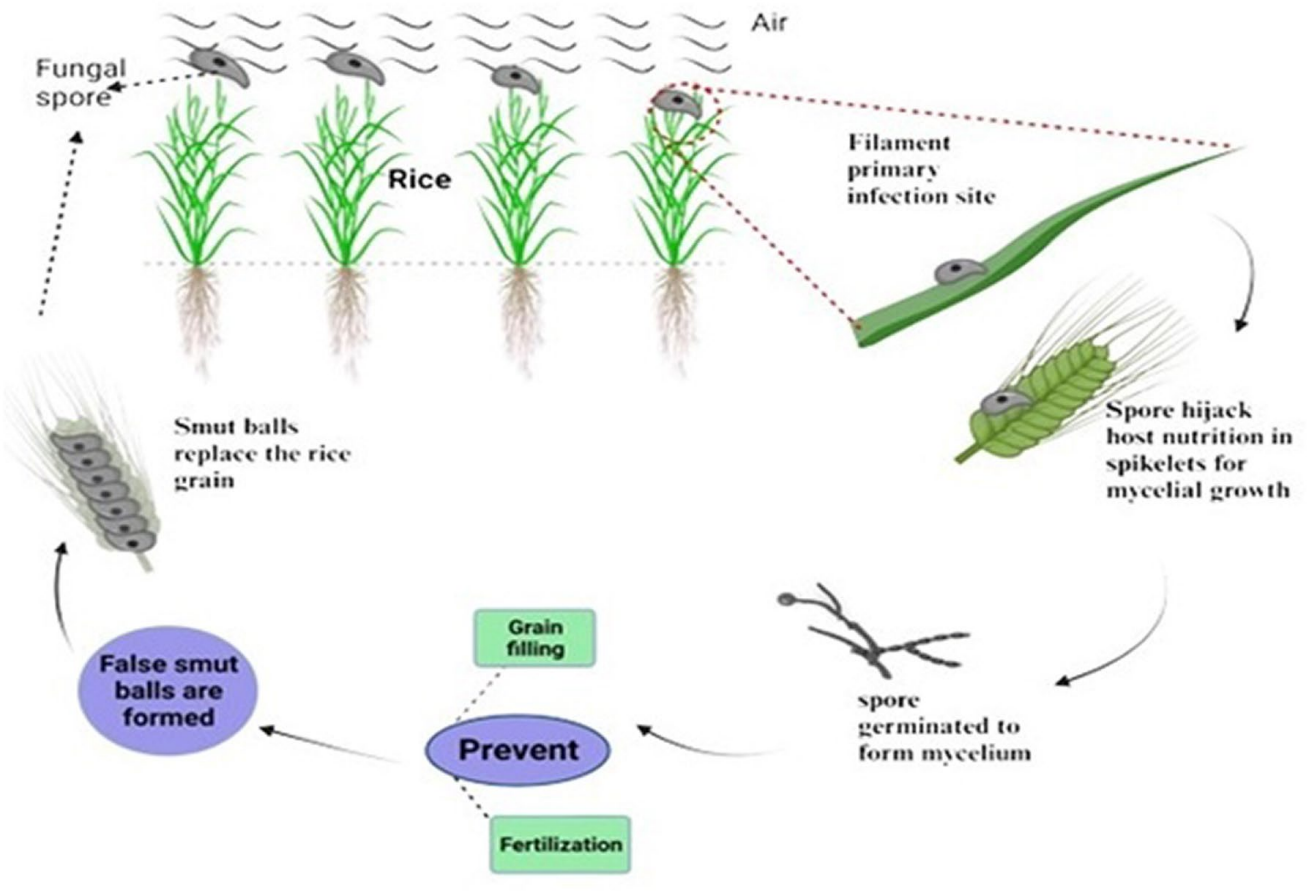
Host-nutrient acquisition and formation of false smut balls.

Mycotoxins are always found in necrotrophic pathogens to damage the host cells and enter the host cell walls with infection hyphae as they extend. It has been observed that the rice false smut pathogen contains significant mycotoxins. However, the filament cells and ovaries remained in newly matured balls (Tang et al., 2013). Most common biotrophic and hemibiotrophic plant pathogens, including rust and powdery mildew, enter host cells and develop particular organs called haustoria that absorb nutrients. No typical infective hyphae or haustoria, which could penetrate host cell walls and enter the host cell through a hole, were found during the infection and development of rice false smut. This suggests that *U. virens* is a biotrophic parasite with a unique mechanism for nutrient acquisition. The peculiar infection mechanism of *Ustilaginoidea virens* appears to be a biotrophic pathogen that also produces mycotoxins. Rice false smut (RFS), one of the most destroying grain diseases in staple crops, has been brought on by the ascomycete fungus *Ustilaginoidea virens* (Cook) Takahashi (teleomorph: *Villosiclava virens*). During the booting stage, *U. virens* spores land on rice spikes and leaves, subsequently given the right conditions to germinate (Raju et al., 2020). Spore-forming hyphae infect rice florets through stamen filaments and reach the inner space of spikelets through tiny gaps between the lemma and palea. The *U. virens* infection prevents fertilization and grain filling, rerouting host nutrients to encourage the creation of false smut balls (Figure 4). RFS results in blighted grains and a decrease in 1000-grain weight. RFS is becoming more frequent, endangering rice production all across the world. Young roots and coleoptiles are among the other rice organs that *U. virens* can infect without presenting disease symptoms. The pathogen encodes significantly fewer glycoside hydrolases than numerous phytopathogenic fungi, the primary enzymes that break down cellulose and xylan in cell walls. Since stamen filaments lack cell wall components and are loosely oriented, they are more susceptible to pathogen infection, according to studies on the cell wall ultrastructure of rice florets. Because RFS is a rare floret disease, it is possible to study grain illnesses’ invasion and particular interactions using the *U. virens*-rice path system as a model.

## Mycotoxins produced by *Ustilaginoidea virens*

In most countries that produce rice, rice false smut (RFS) is now an emerging and significant fungal disease economically (Neelam et al., 2022). In addition to yield losses, RFS also causes many mycotoxins to be produced in false smut balls, endangering both human and animal health. These mycotoxins include but are not limited to ustiloxins, ustilaginoidins, and sorbicillinoids (Yu et al., 2023). Cyclic peptide derivatives known as ustiloxins are poisonous to both plants and animals. Ustiloxins primarily prevent the development of the cytoskeleton and the assembly of microtubules. According to a recent study, ustiloxins A and B were found in various concentrations in 240 samples of rice from China and 72 samples from 12 other nations (Sun et al., 2022a,b). The potential hazards of ustiloxins to human health are highlighted by the widespread contamination of rice in various geographic areas. The effects of RFS have been thoroughly documented since the 1970s in China’s major planting regions. Since the 1980s, when agricultural practices were altered and hybrid rice varieties were widely promoted, RFS has shown an upward trend every year, and it now negatively affects close to one-third of the area used for rice cultivation. RFS harm has been the subject of ongoing investigation. In addition to the apparent yield loss, it can generate significant quantities of toxins that can contaminate grains. Ustiloxins are the most extensively recognized toxin produced by the RFS pathogen (Huang et al., 2021).

## Ustiloxins

Ustiloxins are a group of cyclopetides containing a 13-membered cyclic core structure with an ether linkage. Cyclic peptides called ustiloxins have been shown to have antimitotic activity by preventing the production of cell skeletons and microtubules in both plant and animal cells. Ustiloxins A, B, C, D, F, and G are the six different types of ustiloxins that have been found and given those names. Among them, ustiloxin A and B the two main types, comprise around 90% of the total amount of ustiloxins found in mature false smut balls (FSBs). According to numerous researches, ustiloxins have been found to be highly poisonous to animals. Domestic animals exhibit a range of symptoms when fed contaminated rice grains or feed for a brief period, with the most common being diarrhea, hemorrhage, vomiting, ovarian atrophy, and miscarriage. Reports of abrupt liver and renal necrosis have been brought on by ustiloxin A and the crude water extract of FSBs. Acute liver and renal necrosis in mice have been linked to ustiloxin A and the crude water extract of FSBs. The most recent toxicology research demonstrates that ustiloxin A can impact early-stage zebrafish development and may decrease the Tetrahymena thermophile population by altering the cell cycle. Ustiloxin pollution has severely endangered food safety as RFS becomes more severe in China.

## Ustilaginoidins

According to reports, a false smut pathogen and rice FSBs can create both mycotoxins ustiloxins and ustilaginoidins. Eighteen of the bis-naphtho-pyrone mycotoxins known as ustilaginoidins, including isochaetochromin B2, ustilaginoidins A through P, and ustilaginoidin E1, have so far been discovered. Inhibitory effect on the formation of triacylglycerol in mammalian cells, cytotoxic activity, antibacterial activity, inhibitory activity on HIV-1 integrase, phytotoxic activity, and other biological activities are just a few of the biological traits they display. Ustilaginoidins are derivatives of bis-naphtho-pyrone. Ustilaginoidin D is a possible danger to food safety because it can cause hepatotoxicity and a malfunction of lipid metabolism in zebrafish larvae. All five human cancer cells are cytotoxic to ursulaginoidin M1, although this toxicity is non-specific and prevents the formation of all macromolecules in healthy cells. Therefore, further research is needed to determine whether ustilaginoidin M1 can be employed as a cancer treatment.

## Sorbicillinoids

Sorbicillinoids have recently been discovered to constitute another class of mycotoxins in *U. virens*. Cell cycle arrest and apoptosis are brought on by sorbicillinoids, which also have phytotoxic and antibacterial properties (Hou et al., 2022). In fungi, there are many different sorbicillinoids. Since 2016, 69 sorbicillinoids have been identified (Hou et al., 2022).

## Detection methods of *Ustilaginoidea virens*

Mycotoxin detection methods are crucial for the analysis of agricultural products. Instrumental analysis detection and immunoanalytical detection are currently the primary mycotoxin detection techniques (TeBeest et al., 2011). The main applications of instrumental analysis are in laboratories, businesses, and farming operations. It usually requires expensive equipment and highly qualified experts but is always accurate and reliable. Immunoassays are frequently utilized for on-site rapid detection in food security, and research into the rapid immunological detection of small molecule targets has advanced significantly in recent years. There has been a new understanding of the mechanisms governing antibody stability and affinities (Goding, 1996). Numerous novel antibodies have been created, including nanoantibodies and antibodies created through molecular engineering. Several quick detection tools and associated analytical techniques have been developed, including optical and electrochemical immune sensors, novel immune kits, and test paper strips. Six different types of ustiloxins, of which ustiloxin A is the primary one, have been isolated from false smut balls (FSBs). The toxins can damage people and animals and stunt the growth of grains, including rice, wheat, and maize. However, except for that in mature FSB, there have not been many investigations on the content of ustiloxin. It is unclear how ustiloxins affect the progression of infection. Due to the potential health hazards, ustiloxin A (UA) and ustiloxin B (UB), two primary mycotoxins produced by the pathogen of RFS during rice cultivation, have come under more attention. However, there is a shortage of information about their incidence, fate, and rice contamination profiles. An investigation on the prevalence and translocation of UA and UB in RFS-occurred paddies was conducted in a field.

The two ustiloxins were found in paddy water (range: 0.01–3.46 g/L for UA and 0.02–1.15 g/L for UB) and brown rice (range: 0.09–154.08 g/kg for UA and 0.09–23.57 g/kg for UB) for the first time. Ustiloxin levels in paddy water and brown rice were shown to be significantly correlated with one another (rs = 0.48–0.79, p 0.01). The rice exposure experiment also demonstrated ustiloxin uptake in the water-rice system, indicating paddy water may be a significant source of ustiloxin accumulation in rice. As evidenced by the very high detection rates of UA (96.6%) and UB (62.4%) in polished rice (149 samples) from Hubei Province, China, these results suggested that ustiloxin contamination of rice might occur frequently. In the polished rice samples from Hubei Province, ustiloxin contents ranged from 20.7 ng/kg (LOD) to 55.1 g/kg (dry weight). More research is required to assess the possible dangers of ustiloxin exposure in the environment and in humans. Due to their high hydrophilicity, ustiloxins can enter the surrounding environmental matrix by air deposition or rain. Ustiloxin contamination, for instance, was discovered to be pervasive in the water of RFS-occurring paddies, with values as high as 2.82 g/L. In areas with RFS, rice plants including uninfected plants should be exposed to ustiloxins on a large scale. Ustiloxins might be absorbed by rice plant roots from contaminated paddy waters in paddy fields where RFS occurred. More significantly, the presence of high quantities of ustiloxins in polished rice samples suggests that rice plants may be able to take in ustiloxins from the environment. Residual ustiloxins can be absorbed and deposited in rice plants, leading to widespread contamination of rice grains. However, no research has been performed to investigate rice plants’ uptake, bioaccumulation, and translocation of ustiloxins. The rice false smut pathogen (Ustilaginoidea virens) can produce mycotoxins that can harm rice plants in several ways (Wang et al., 2019). A fungal disorder called rice false smut can produce mycotoxins, hazardous secondary metabolites. These mycotoxins may impact the health and yield of rice plants in several ways. RFS has reached worldwide importance, particularly when hybrid rice growing dominates the production acreage. Regarding exporting and producing hybrid rice seeds, China leads the globe in both categories. Therefore, before exporting the seeds, it is crucial to devise a straightforward technique for quickly identifying this disease. PCR has been a commonly used technique for disease detection in plant seeds because of its accuracy and practicality (Hension and French, 1993). Chen et al. (2014) established a quick and easy one-step PCR method for detecting *U. virens*. The detection may be completed in less than three hours, and the methodology works well when examining many rice seed samples. Using this technique, first verified that the PCR product size of 346 bp fragment, which is lacking in the other examined fungus and the negative control, was amplified by the chosen primers. Furthermore, rather than using fungal DNA, the same procedure was validated using the seed wash supernatant (following centrifugation). As a result, many samples may be screened quickly using the devised technique. Therefore, even if the established technique might be more precise, it would significantly increase the detection efficiency when compared to the nested PCR, which requires the execution of two PCR cycles (Zhou et al., 2003). The ability to handle vast samples of rice seeds more effectively will be aided by developing a quick and easy method for detecting *U. virens*.

## Reduced plant growth and yield

The rice false smut pathogen produces mycotoxins that can limit plant growth and development, resulting in a decreased yield. They may interfere with several physiological processes, including hormone control, nutrient uptake, and photosynthesis, ultimately affecting plant growth and grain production. Impaired Nutrient Uptake: Mycotoxins may prevent plants from properly absorbing vital nutrients from the soil. Nutrient deficits may occur, harming plants’ general health and productivity.

## Toxicity

The rice false smut pathogen can produce several mycotoxins that are directly hazardous to rice plants. Cell death and tissue necrosis may result from the damage they cause to plant cells and tissues. The plant’s structural integrity may be compromised, making it more vulnerable to various illnesses and environmental stresses. Hormonal Disruption: Mycotoxins can potentially disrupt the hormonal balance in rice plants. Hormones are essential for controlling many physiological processes, and when this balance is upset, abnormal growth patterns, developmental anomalies, and decreased yield might result. Rice grain quality may suffer as a result of mycotoxins. Mycotoxins can make grains unfit for processing or consumption, which could impact food security and cause farmers to lose money.

## Environmental stress

Rice plants exposed to mycotoxins and infected with the false smut pathogen may face more environmental stress. They may be more vulnerable to various illnesses, pests, and unfavorable environmental factors due to this stress. It’s crucial to remember that the precise impacts of mycotoxins on rice plants might differ based on several variables, including the type and quantity of mycotoxins produced, the type of rice grown, the environment, and agricultural practices. Fungicide use, crop rotation, and resistant types are some effective disease management techniques that can lessen the detrimental impacts of mycotoxins.

## Management of rice false smut

Reducing the severity of the disease depends on an integrated approach to disease management. To avoid and control the illness, it begins with selecting seeds, considering appropriate cultural techniques, and the choosing an efficient control agent. By lowering disease infestation, optimal consideration of preventative and control strategies at the proper times will increase yield.

## Preventive approaches

### Resistance varieties

There are variations in the occurrence and intensity of smut among cultivars planted in the same location or area (Kiran et al., 2021; An et al., 2023). In certain cultivars, lower infection levels have been reported (Khanal et al., 2023). Numerous workers have observed that many rice types are tolerant or resistant based on how they respond in fields under natural conditions. Kaur et al. (2015) screened 125 rice genotypes by artificial inoculation and identified nine hybrids (KRH-4, Hybrids VNR-211, 27P64, IRH-74, RH-10428, PRSH-9018, GK-5025, KPH-467, and HRI-140) shown complete resistance.

### Cultural practices

Studies carried out in the United States demonstrated that a number of alternative crop management techniques, together with soil tillage, crop rotation, and fertility rate, might effectively suppress smuts in sensitive rice cultivars (Khanal et al., 2023). The same researchers had previously discovered that in vulnerable cultivars, the moderate application of nitrogen fertility rates decreased false smut disease (Das et al., 2024). When compared to late planting, the disease incidence was greater in early transplanted rice (Leharwan and Kumar, 2023). Planning the planting date and heading period so blossoming does not coincide with a rainy season will help prevent serious damage. Farmers may be able to lessen the disease’s early onset by using sclerotic-free seeds for bund cleaning and sowing.

## Control approaches

### Chemical method

The growth of fungal mycelium was completely inhibited by the fungicides Triûoxystrobin 25%+, Tebuconazole 50%, and Propiconazole 25 EC when tested *in vitro* and *in vivo*. The fake smut of rice was effectively controlled by applying prochloraz + carbendazim and then chlorothalonil (Mohiddin et al., 2012). Nine modern fungicides were tested in a 2016 kharif trial against the false smut disease that affects rice. Azoxystrobin (18.2%), SC + Difenconozole (11.4%), and Metiram (55%) were the various fungicides that were tested. The lowest illness severity was reported by WG + Pyraclostrobin (5%) WG @ 0.1%, with 1.85 and 2.52 percent, respectively. Propiconazole 25 EC, Azoxystrobin 25% SC, Difenconazole 25% EC, Tebuconozole 250 EC, and Flusilazole (25%) were the next in line. Carbendazim + SE (12.5%) Under field conditions, SE increased the paddy yield and demonstrated improved efficacy at 0.1% (Muniraju et al., 2017). According to Raji et al. (2016), whether sprayed at booting or 50% panicle emergence, propiconazole 25EC (0.1%) recorded the lowest disease severity compared to other treatments. Trifloxistrobin + Tebuconazole 75 WG came in second. Spraying Trifloxystrobin + Tebuconazole 75 WG and Propiconazole 25 EC produced higher yields during the booting stage.

## Plant extracts and essential oils

Plant extracts were studied *in vitro* against the rice false smut pathogen by Raji et al. (2016). The plant extracts of garlic bulb (*Allium sativum*), turmeric rhizome extract (*Curcuma longa*), lantana leaf extract (*Lantana camara*), and bael (*Aeglemarmelos*) significantly inhibited the growth of *U. virens*, while the plant oils of lemon grass (*Cymbopogon flexuous*), cinnamon (*Cinnamomum zeylanicum*), and palmarosa (*Cymbopogon martinii*) completely inhibited the growth of *U. virens*.

## Biocontrol agents

*Antennariella placitae* has been shown to be effective against false rice smut in both *in vitro* and *in vivo* conditions by Andargie et al. (2017). Nine isolates of *Trichoderma viride*, *Trichoderma virens, Trichoderma harzianum*, and *Trichoderma reesei* were obtained from rice rhizosphere and studied for their antagonistic potential *in vitro* by Kannahi et al. (2016). They found that all of the isolates of Trichoderma showed antagonistic activity against *U. virens*, but that the isolate of *T. viride* had the highest antagonistic potential.

## Conclusion and future prospects

Until recently, rice false smut was considered one of the minor diseases of rice, but recently, cases of the disease have increased across all of the leading rice-growing nations. Nearly a century of disease-related study has given us a detailed understanding of the disease and some practical solutions for reducing its spread. False smut is easily distinguished by its smut ball look on the rice grains, which range in color from yellow to dark green. These smuts are poisonous to people and can hurt their health. Studies link the occurrence of the disease to factors such as rain during blooming, greater nitrogen use, poor cultural practices, and susceptible varieties. To lower disease prevalence, one could choose to apply chemicals or not. The use of disease-resistant varieties like VNR-211, GK-5025, HRI-140, and others along with cultural practices like timing paddy transplants to avoid rain during the reproductive stage, applying the right amount of nitrogen, and rotating crops, all work together to reduce the disease’s occurrence organically. Chemicals can be utilized to get a quick reaction, and they often have a better reputation for controlling diseases. Numerous scientific studies in the field of agricultural development want to be strengthened. All desirable kinds of paddy do not possess the ability to withstand paddy fake smut. The majority of the research in these fields has produced methods for bringing the sickness down to a minimal. The pathologist, agronomist, and breeder must determine how to address the growing environments. Introducing a resistant gene to the popular hybrid paddy will be a significant relief to the crop-growing regions. Additionally, while producing seeds for organic gardeners, consideration must be paid to selecting resistant lines. Scientists working together in the region to study diseases will be able to provide consumers safe food and crop growers with a customized solution.

## Limitations

The metabolic alterations brought about by infection in the filaments and why this target tissue is particularly vulnerable to pathogen attack will be the focus of more research? Has any study been performed to investigate the uptake, bioaccumulation, and translocation of ustiloxins in rice plants? More research is required to assess the possible dangers of ustiloxins exposure in the environment and in humans.?

## Author contributions

LZ: Conceptualization, writing – review & editing. MM: Data curation, writing – original draft, writing – review & editing. YI: Investigation, writing – review & editing. HZ: Funding acquisition, writing – review & editing. ZZ: Resources, writing – review & editing. JW: Visualization, writing – review & editing. RAAK: Methodology, writing – review & editing. AS: Writing – review & editing. MKS: Project administration, writing – review & editing. MAS: Software, writing – review & editing. AK: Formal Analysis, writing – review & editing. EESM: Validation, writing – review & editing. LC: Supervision, Writing – review & editing.
